# How Do Different Growth Forms of Winter Submerged Macrophytes Species Respond to Underwater Light Quality in a Mesocosm Study?

**DOI:** 10.1002/ece3.70441

**Published:** 2024-10-18

**Authors:** Xiaowen Lin, Xiaodong Wu, Xuguang Ge, Chenxin Zhong, Zian Xiang, Ye Yao, Lishuai Zhang, Sizhuo Li

**Affiliations:** ^1^ College of Urban and Environmental Sciences Hubei Normal University Huangshi China; ^2^ Resource‐Exhausted City Transformation and Development Research Center Hubei Normal University Huangshi China

**Keywords:** aquatic restoration, *Elodea nuttallii*, growth form, light quality, *Potamogeton crispus*

## Abstract

Underwater light is a key factor that affects the growth of submerged macrophytes. However, the responses of different growth forms of submerged macrophytes to light quality remain unclear. The morphological, physiological, photosynthetic, and stoichiometric responses of erect *Potamogeton crispus* (*P. crispus*) and low‐canopy *Elodea nuttallii* (*E. nuttallii*) to six different light qualities (white light, R/B = 1:8, 1: 4, 1:1, 4:1, 8:1) were studied by a control experiment. (1) No significant differences were observed in the germination number, leaf length, and leaf width of *P. crispus* under different light qualities (*p* > 0.05). Both *P. crispus* and *E. nuttallii* produced greater plant heights, more leaves and branches under more red light (4:1, 8:1), which was beneficial for the extension of leaves. Under white light, the germination number of *P. crispus*, adventitious roots of *E. nuttallii*, and branch number of *P. crispus* and *E. nuttallii* were the lowest. (2) Compared to red light, more blue light (1:4, 1:8) was more conducive to the synthesis of photosynthetic pigments. However, excessive blue and red light was not conducive to the accumulation of pigments. The result of malondialdehyde showed that the physiological stress induced by blue light in *P. crispus* and *E. nuttallii* was stronger. (3) The N:P, C:N, and C:P ratios of *P. crispus* and *E. nuttallii* were higher under more red light, which was beneficial for the synthesis of nutrients in two submerged macrophy; however, the contents of TN and TP in *E. nuttallii* were higher under more blue light. Two different growth forms of submerged macrophytes grew better under red light, and better adapted to the eutrophic water dominated by red light. However, for aquatic restoration, other submerged macrophytes need to be supplemented after the water quality is improved.

## Graphical abstract

The responses of erect *Potamogeton crispus* (*P. crispus*) and low‐canopy *Elodea nuttallii* (*E. nuttallii*) to six different light qualities (white light, R/B=1:8, 1:4, 1:1, 4:1, 8:1) were studied by a control experiment. The two different growth forms of submerged macrophytes both grew better under more red light (4:1, 8:1), and better adapted to the underwater light environment of eutrophic water dominated by red light. Compared to red light, more blue light (1:8, 1:4) was more conducive to the synthesis of photosynthetic pigments. Both *P. crispus* and *E. nuttallii* can be used in the treatment of eutrophic water dominated by red light, but other submerged macrophytes need to be supplemented after water quality improvement.

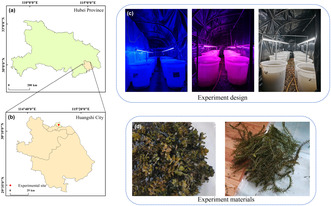

## 1 Introduction

The growth forms of submerged macrophytes can be divided into canopy, rosette, and erect (Gopal and Goel 1993). The canopy form can be divided into high‐ and low‐canopy forms according to height. High‐canopy submerged macrophytes form a large canopy on the water surface, which provides unique advantages for obtaining light and oxygen. However, the canopy will hinder the irradiation of sunlight, which is not conducive to the growth of organisms in the lower space of the water column (Gao et al. 2021; Lin et al. 2023). The biomass of the erect submerged macrophytes is evenly distributed at each depth of the water column. In aquatic ecosystems, because of the niches occupied by different growth forms of submerged macrophytes in the water layer, their coexistence can allow for full use of resources in limited spaces and improvement of the stability of the ecosystem, which is conducive to maintaining the lake in a clear‐water state (Liu et al. 2020; Li et al. 2023). However, different growth forms of submerged macrophytes respond differently to environmental changes (Chen et al. 2020; Yu et al. 2022). Light environment (light intensity and light quality) is an important factor affecting the growth of submerged macrophytes (Yu et al. 2021; Gao et al. 2023; Wang et al. 2022). A good underwater light environment is conducive to stable submerged macrophyte communities, and submerged macrophytes help a lake maintain a clear‐water state. Positive feedback occurs between the underwater light environment and submerged macrophytes (Wang et al. 2019; Su et al. 2019; Liu et al. 2020). When the light intensity is limited, the canopysubmerged macrophytes primarily grow rapidly to the water surface by elongating their stems to obtain sufficient light, while the rosette‐submerged macrophytes adapt to the low‐light environment at the bottom of the water by enhancing their photosynthetic capacity (Liu et al. 2020; Tian et al. 2023). However, the responses of different growth forms of submerged macrophytes to light quality remain unclear now.

In summer, the water quality in most lakes is relatively clear because of the vigorous growth of submerged macrophytes. However, in winter and spring, due to the decline of submerged macrophytes, the release of nutrients from plant residues causes secondary pollution, and the purification function of submerged macrophytes on water bodies cannot be exerted. This often leads to the deterioration of water quality in winter and spring, which in turn causes changes in the underwater light environment (Kang, Xu, and Zou 2020; Liu et al. 2022). Therefore, to improve water quality in eutrophic lakes, it is important to study the different growth forms of submerged macrophytes that can survive in winter under different light qualities. In aquatic ecosystems, in addition to submerged macrophyte communities, there are also multiple communities (such as phytoplankton) that interact with water trophic conditions and underwater radiation (Zhang et al. 2023). Eutrophication levels tend to be higher in waters dominated by cyanobacteria, whereas nutrient‐rich waters contribute to the proliferation of phytoplankton (Mackay et al. 2020; Zhang et al. 2023). In eutrophic waters, due to the higher content of dissolved organic matter and particulate matter, blue light also attenuates faster and the ratio of red to blue light (R/B) increases (Zhang et al. 2005; Lin et al. 2024). The deterioration of the underwater light environment in turn affects the community composition and diversity of phytoplankton, leading to damage to the food chain, and is conducive to the invasion of aquatic alien plants (Huang et al. 2023). Due to the complex community composition of aquatic ecosystems, there are dynamic changes in biological components in ecosystems. Therefore, the underwater light quality in lakes also changes significantly during different seasons. Red and blue light attenuates the slowest in winter, resulting in the lowest R/B ratios in winter (Liu et al. 2020; Lin et al. 2024). In winter, when growth rates are low, artificial light (red, blue, and white light) supplementation promotes the growth and recovery of submerged macrophytes (Xu et al. 2019). The effect of ultraviolet (UV) radiation on the growth of *Potamogeton crispus* (*P. crispus*) has been previously investigated (Wang et al. 2015, 2016; Wang, Song, and Xue 2015), and the appropriate combination of red and blue light has been shown to be more conducive to plant growth, photosynthetic pigment synthesis, and carbon (C) and nitrogen (N) metabolism than monochromatic light (Di et al. 2020). However, there remains a gap in our understanding of the effects of different R/B ratios on submerged macrophytes during winter.

*P. crispus* is a widely distributed submerged plant in the world. It grows in the north and south provinces of China, especially in the Yangtze River Basin (Chen, Ma, and Du 2012). *P. crispus* is the dominant species in many eutrophic lakes and can be used for water purification from winter to early summer when other submerged macrophytes decline (Wang, Song, and Wang 2017; Liu et al. 2022). *P. crispus* is an erect submerged plant. We selected another winter submerged plant with a different growth form: the low‐canopy form *Elodea nuttallii* (*E. nuttallii*). The origin of *E. nuttallii* is in the temperate zone of the Americas. In the 1980 s, it was first introduced from Japan to Taihu Lake in China and then spread widely (Xu et al. 2019). In this study, we aimed to explore how *P. crispus* and *E. nuttallii* respond to light quality and under which light quality they grow better in terms of morphological, physiological traits, and stoichiometry. In natural waters, blue light often attenuates faster than red light (Zhang et al. 2005; Lin et al. 2024). So red light is often dominant at the bottom of the water body. *E. nuttallii* is a low‐canopy submerged plant, mainly growing in the lower space of the water column. Therefore, we hypothesized that *P. crispus* and *E. nuttallii* will have different responses under different light qualities due to growth forms. *E. nuttallii* may grow better under more red light, while *P. crispus* is an erect submerged plant, which may grow better under more blue light. Our study can provide a scientific basis for the restoration of submerged macrophytes in lakes with different nutritional levels during winter.

## 2 Materials and Methods

### 2.1 Experimental Materials

Both *P. crispus* and *E. nuttallii* can grow during winter. *P. crispus* is a perennial submerged herb belonging to the genus *Potamogeton* in the family *Potamogetonaceae*. Its stem is cylindrical and multi‐branched. The leaves are all submerged leaves, wide strip or strip lanceolate, and the leaf margin has wavy folds. Inflorescences are spike‐shaped. The flowering and fruiting period is from April to July. It has strong adaptability to eutrophic waters but not high temperature resistance (Chen, Ma, and Du 2012). The reproduction of *P. crispus* primarily depends on turions. Unlike most submerged macrophytes, *P. crispus* sprouts in autumn and grows during the winter. In summer, *P. crispus* declines and dies. Turions of the same size, weight, health, and vitality were selected for the experiment.

*E. nuttallii* belongs to *Elodea* of *Hydrocharitaceae*. The texture of *E. nuttallii* is brittle. The leaves are often three‐impeller‐shaped and have purple or black spots. The edges of its leaves are serrated. Inflorescences are solitary, and without pedicels. Flowering and fruiting period occur from July to October. *E. nuttallii* can survive in winter with vegetative propagules and has strong vitality. It can survive in an environment with no ice and grow when the temperature above 4 ± 1°C (Kunii 1982, 1984). *E. nuttallii* with apical tips, no adventitious roots, and no branches were selected as the experimental materials. *E. nuttallii* were washed with clean water and cut to a length of 15 cm using scissors. In this experiment, *P. crispus* and *E. nuttallii* were purchased online.

### 2.2 Experimental Design

**FIGURE 1:**
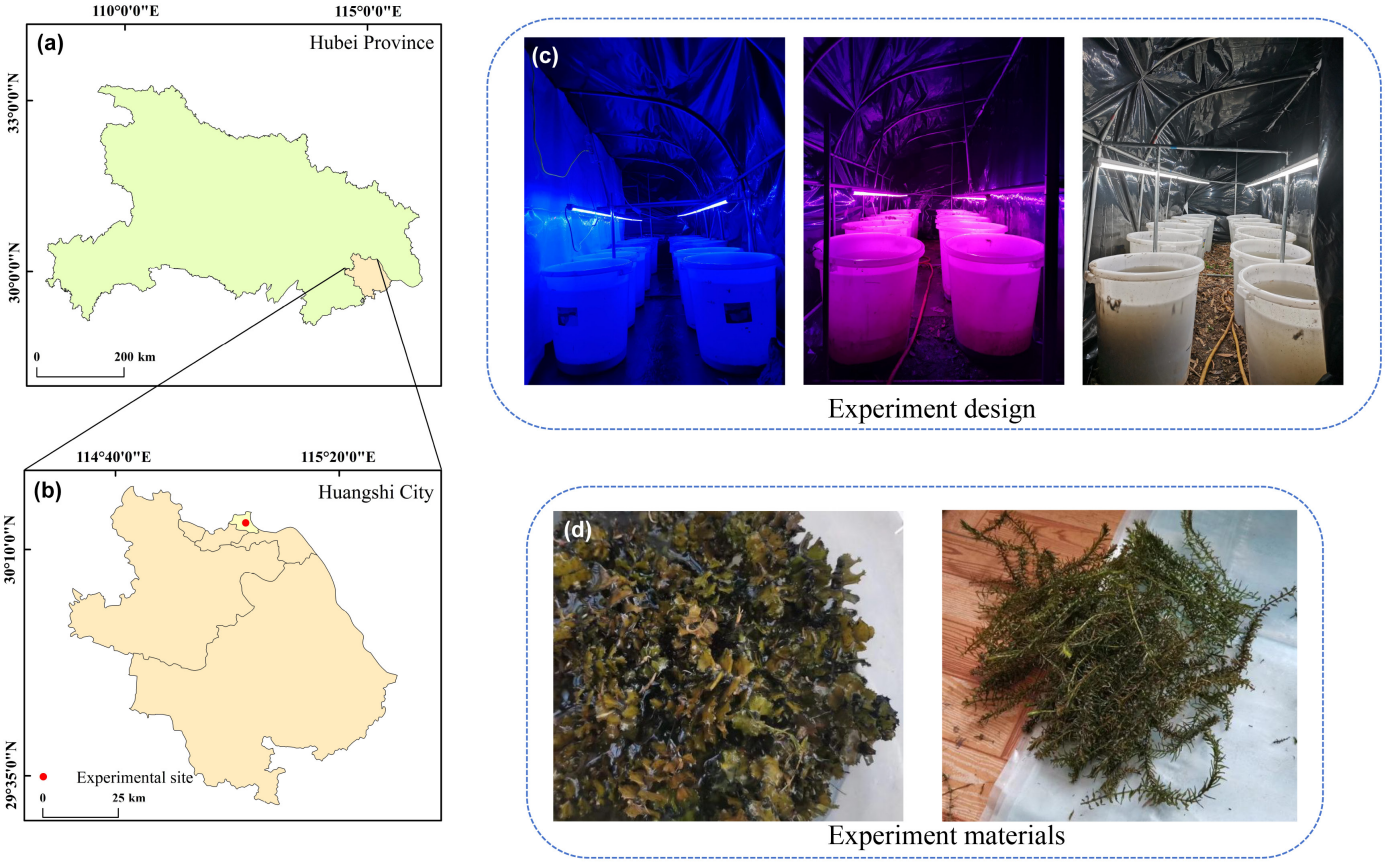
Study area (a, b) and experimental design (c, d). Figure 1 (c) was taken after planting and then just adding water. When water was added, the water inevitably causes some disturbance to the sediment, so the water looks somewhat turbid. In fact, our experiment began after the water was clear.

**FIGURE 2:**
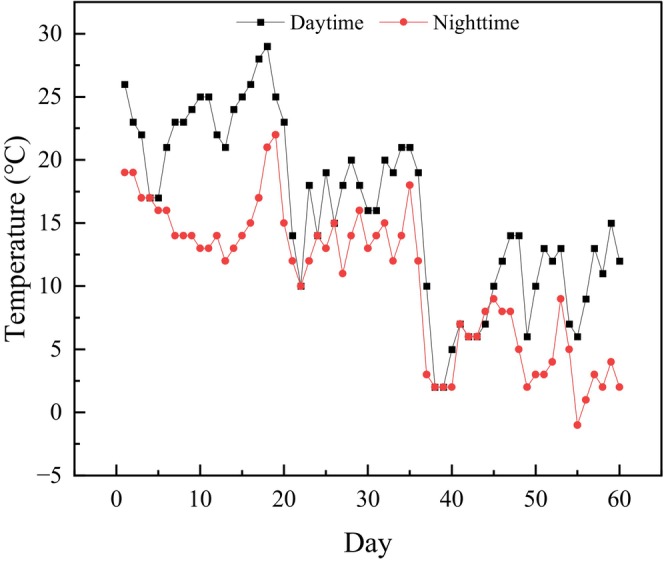
Temperature changes during the experiment.

To exclude the interference of natural light, the experiment was carried out in a black‐and‐white plastic film greenhouse (115° 3′ 17″ E, 30° 14′ 7″ N) near Qingshan Lake in the middle reaches of the Yangtze River in China (Figure 1). The experiment began on October 24, 2022, and ended on December 22, a total of 60 days. The daytime and nighttime temperatures were measured by a thermometer at 12:00 and 23:00 during the experiment, respectively (Figure 2).

The sediment of Qingshan Lake was added to a plastic barrel with an inner diameter of 57 cm and a height of 60 cm. The volume of plastic barrel is 120 L. The sediment was filtered through a sieve to remove impurities such as snails, shellfish, and stones. Then mixed the sediment and added them to each plastic barrel after mixing. We take a portion of the sediment from the fully mixed sediment. After natural drying, grinding and passing through a 100‐mesh sieve, total phosphorus (TP), total nitrogen (TN), organic matter (OM) in the sediments were determined. TN and TP in sediment were determined using alkaline potassium persulfate digestion and potassium persulfate oxidation method, respectively. OM was determined using the potassium dichromate volumetric method (Wang 2014). In our experiment, TP, TN, and OM in the sediment were 1.45 g/kg, 0.29 g/kg, and 30.3 g/kg, respectively.

Sixteen *P. crispus* turions and 10 *E. nuttallii* were evenly planted in concentric circles. When planting *P. crispus* turions, they were gently pressed into the sediment to ensure that the entire turion was just submerged in the sediment. After planting, tapwater (in order to eliminate the influence of algae in water [Li et al. 2015]) was gently added to the barrel up to a height of 50 cm. We have taken measures to avoid the disturbance of water flow to the sediment to the greatest extent when we added water. However, it still inevitably caused some slight disturbances to the sediments. In order to reduce the influence of turbidity on light transmittance, we began our experiment after the water was clear. TN (0.82–1.34 mg/L) and TP (0.031–0.039 mg/L) of the water were measured. To ensure a consistent water level and reduce the influence of water evaporation, water was replenished in the plastic barrel over time during the experiment.

Each of the two plastic barrels share one LED tube. Therefore, each treatment group was combined in series with three LEDs. LED tubes were installed above plastic barrels in each treatment group. LED tubes were composed of many bulbs. In the 1:8, 1:4, 1:1, 4:1, and 8:1 treatment groups, the LEDs were composed of different ratios of red (R) to blue (B) bulbs, and white light was used as the control group (CK). There were six replicates for each light quality and a total of 72 barrels (six light qualities × six replicates × two species). The light intensity of each LEDs was about 100 μmol/m^2^/s, and the illumination time was 7:00–19:00 (simulating the day and night cycle in the natural state).

### 2.3 Measured Indicators

#### 2.3.1 Morphological Indicators

*P. crispus* was planted as turions, and the number of germinated turions in each treatment group was observed and recorded every 2 days. Individuals exposed to the sediment surface and visible to the naked eye were considered germinated. Leaf lengths and widths of *P. crispus* were measured on days 30 and 60. Plant height, stem node number, leaf number, and branch number of *P. crispus* and *E. nuttallii* were recorded every 10 days. Plant height was measured by tape. The number of stem nodes, leaves, and branches was measured by manual counting. The number of adventitious roots (the roots that were in the water but not buried in the sediment) of *E. nuttallii* was recorded every 20 days. The root–shoot ratio (R/S) of *P. crispus* and *E. nuttallii* was measured at the end of the experiment. The fomula is as follows (Li et al. 2015):

$$\frac{R}{S}=\frac{w_{1}}{w_{2}}$$

where *w*_1_ is the underground biomass (g) and *w*_2_ is the aboveground biomass (g).

#### 2.3.2 Physiological Indicators

1. Chlorophyll (Chl)

Fresh plant leaves (0.2 g) were weighed for each treatment group on day 60, and the pigment was extracted using 95% ethanol. The detailed steps are referred to in the “Plant Physiology Experiment Tutorial” (Wang 2017). Absorbance was measured at 665, 649, and 470 nm by an ultraviolet spectrophotometer, and chlorophyll a (Chl a) and chlorophyll b (Chl b) contents were calculated as follows:

$$\begin{array}{l} \text{Chl}\;\text{a}=13.95\times A_{665}-6.88\times A_{649}\\ \text{Chl}\;\text{b}=24.96\times A_{649}-7.32\times A_{665} \end{array}$$

where *A* is the absorbance value.

2 Malondialdehyde (MDA)

MDA levels were determined using the thiobarbituric acid method (Wang 2017). The absorbance of the supernatants was determined at 450, 532, and 600 nm. The formula is as follows:

$$C\left(\text{μmol}/L\right)=6.45\left(A_{532}-A_{600}\right)-0.56A_{450}$$

where *A* is the absorbance value.

#### 2.3.3 Stoichiometric Indicators

At the end of the experiment, the plants were dried, then ground using a mortar, and passed through a 60‐mesh sieve. A specific amount of plant powder was weighed to determine TP, TN, and total organic carbon (TOC). The contents of TP, TN, and TOC were determined using the ammonium molybdate ascorbic acid method, an automatic N analyzer, and potassium dichromate oxidation spectrophotometry (Bao 2000), respectively. C:N, C:P, and N:P were calculated as follows:

$$\begin{array}{l} C:N=\frac{\mathrm{TOC}}{12}:\frac{\mathrm{TN}}{14}\\ C:P=\frac{\mathrm{TOC}}{12}:\frac{\mathrm{TP}}{31}\\ N:P=\frac{\mathrm{TN}}{14}:\frac{\mathrm{TP}}{31} \end{array}$$

where TN, TP, and TOC are the contents of TN, TP, and TOC, respectively (g/kg).

### 2.4 Data Analysis

All experimental data were processed using Excel. One‐way analysis of variance implemented in Origin 2021 (Origin Labs Inc., Northampton, MA, USA) was used to analyze the differences in morphological, physiological traits, and stoichiometric indices of *P. crispus* and *E. nuttallii* under different light qualities. Tukey's test was used for post hoc multiple comparisons (*p* < 0.05). Before the analysis of all data, the Shapiro–Wilk and Levene methods were used to test normality and variance homogeneity. Principal component analysis (PCA) was used to explore the relationships between the morphological, physiological traits, and stoichiometric indices of the two submerged plants. All figures were created using Origin 2021.

## 3 Results

### 3.1 Morphological Traits

#### 3.1.1 Germination Number

**FIGURE 3:**
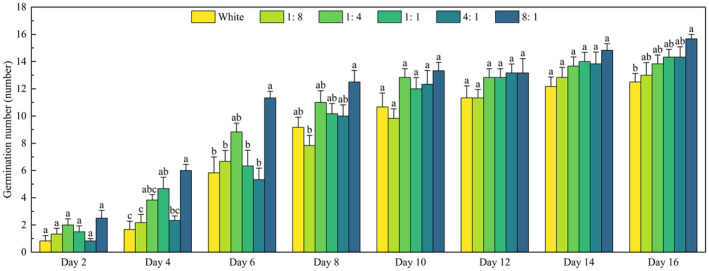
Effects of light quality on the germination number of *P. crispus* turions.

The germination rate of *P. crispus* turions under 8:1 light quality was the fastest and highest (Figure 3), reaching 15.65% on day 2, and the final germination rate was as high as 97.9%. The germination number of the turions increased with the increasing R/B ratio. Under white light, the germination number was the lowest, but it was only significantly lower than that under 8:1 light quality (*p* < 0.05).

Error bars represent standard error. Different lowercase letters represent differences in the germination number of *P. crispus* turions under different light qualities (*p* < 0.05).

#### 3.1.2 Plant Height

**FIGURE 4:**
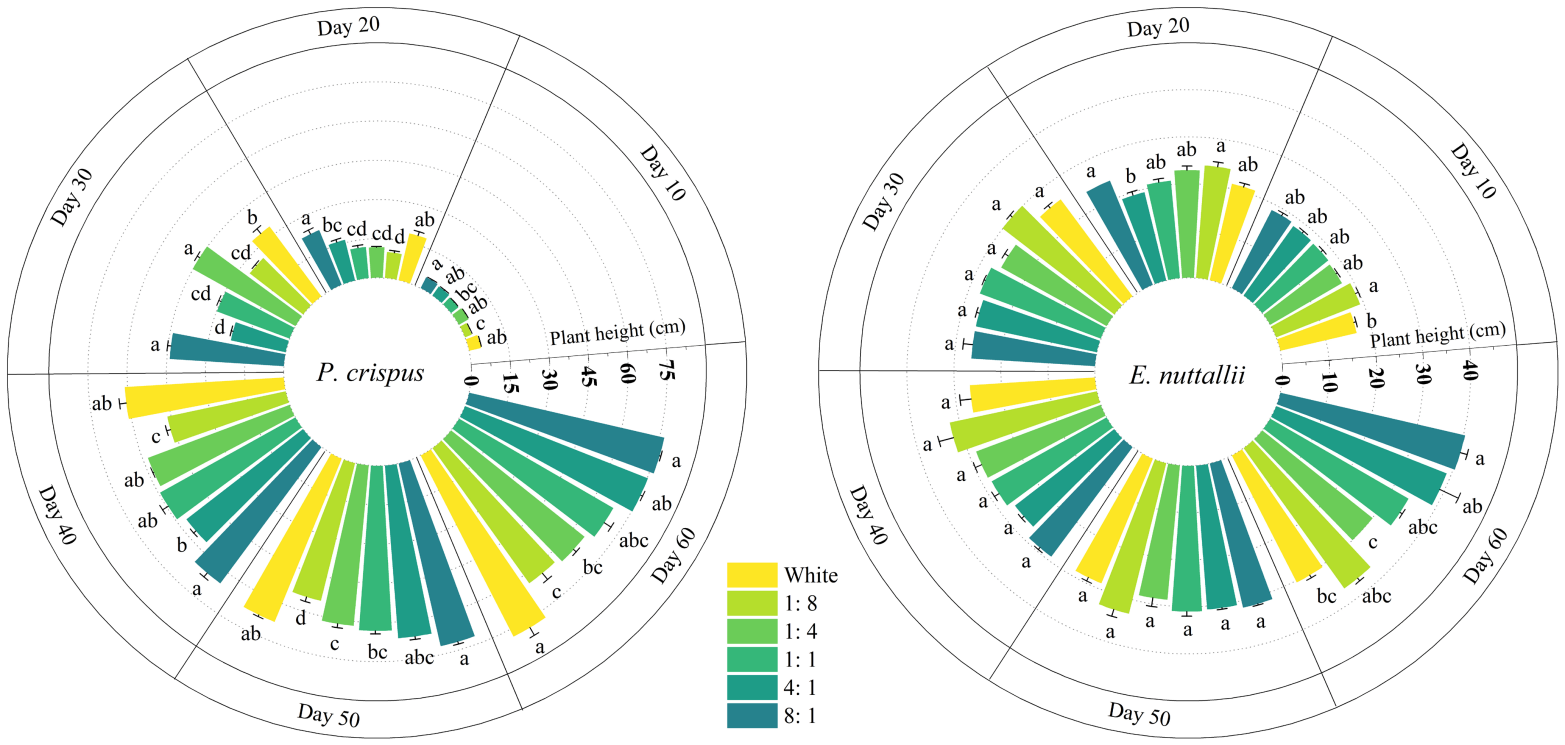
Effects of light quality on plant height of *P. crispus* and *E. nuttallii*.

*P. crispus* and *E. nuttallii* were significantly higher under more red light (4:1, 8:1) than under more blue light (1:8, 1:4) (*p* < 0.05) (Figure 4). The plant height of *P. crispus* increased with an increase in R/B, but its plant height was lower than that of CK (78 cm), which was 1.23 and 1.93 times that of 1:8 and 1:4 (*p* < 0.05). The plant height of *E. nuttallii* under the 1:4 light quality was slightly lower than that in the CK (*p* > 0.05), whereas those under other light qualities were higher than those in the CK.

#### 3.1.3 Stem Node Number

**FIGURE 5:**
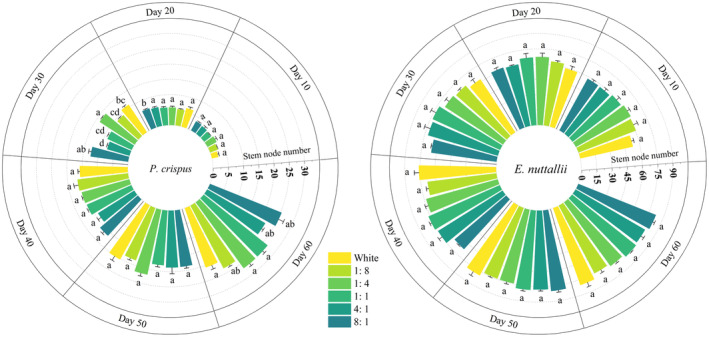
Effects of light quality on stem node number of *P. crispus* and *E. nuttallii*.

The number of stem nodes in *P. crispus* began to differ significantly on day 30 (*p* < 0.05) (Figure 5). At the end of the experiment, the number of stem nodes in *P. crispus* was higher under more blue light (1:8, 1:4) than that under more red light (4:1, 8:1). The effect of light quality on the number of nodes of *E. nuttallii* was not significant throughout the experiment.

#### 3.1.4 Branch Number

**FIGURE 6:**
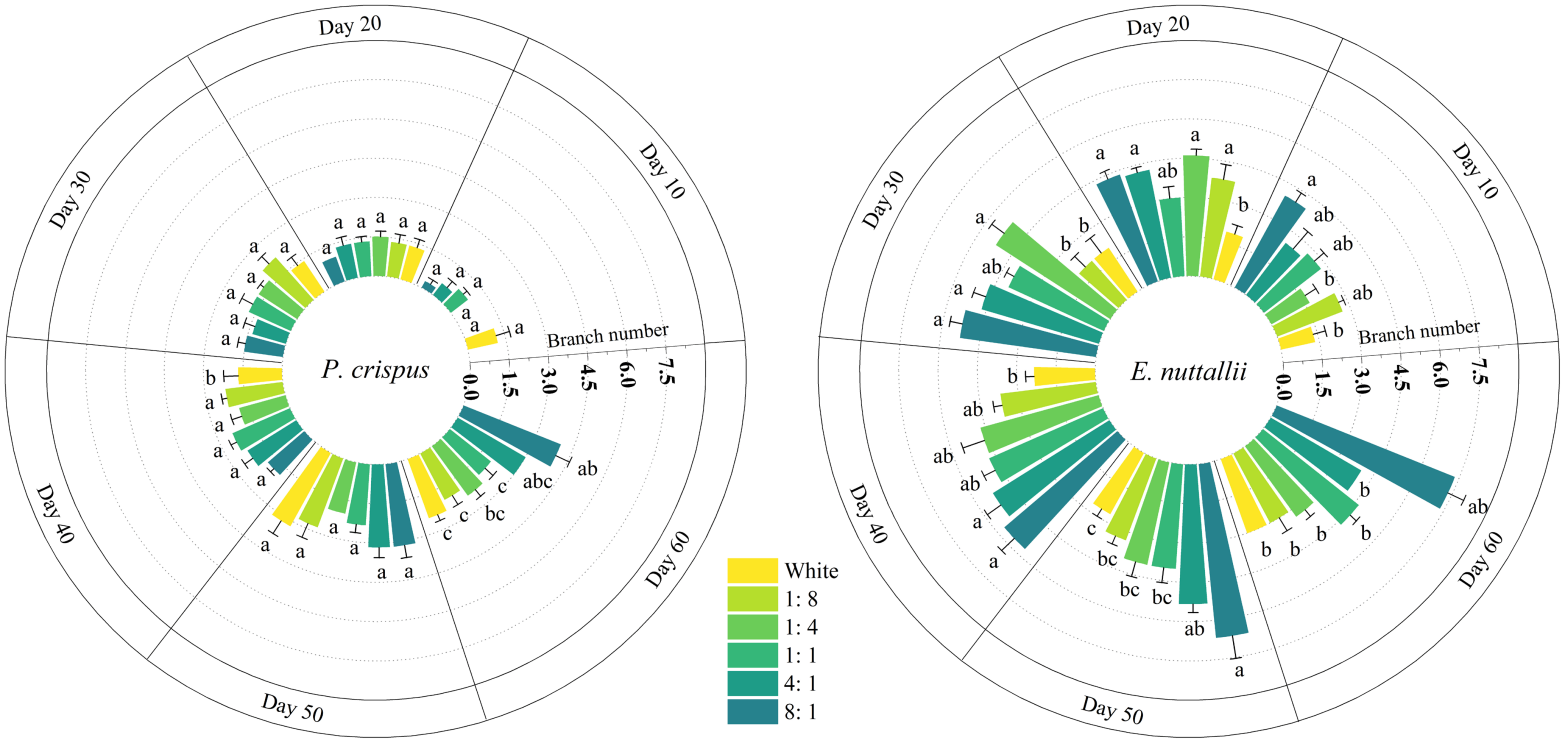
Effects of light quality on branch number of *P. crispus* and *E. nuttallii*.

By day 10, *P. crispus* produced branches (Figure 6), except for those under 1:4 and 1:8 treatment groups. White light produced the most (1.17 branches). However, all *E. nuttallii* under different light qualities produced branches by Day 10, and the number of branches was the highest (3.01 times that of CK) under 8:1 light quality. At the end of the experiment, *P. crispus* and *E. nuttallii* under 8:1 light quality had more branches than those in other treatment groups (*p* < 0.05). Overall, both *P. crispus* and *E. nuttallii* produced more branches under red light (4:1, 8:1). Under the same light quality, *E. nuttallii* had more branches than *P. crispus*.

#### 3.1.5 Leaf Number

**FIGURE 7:**
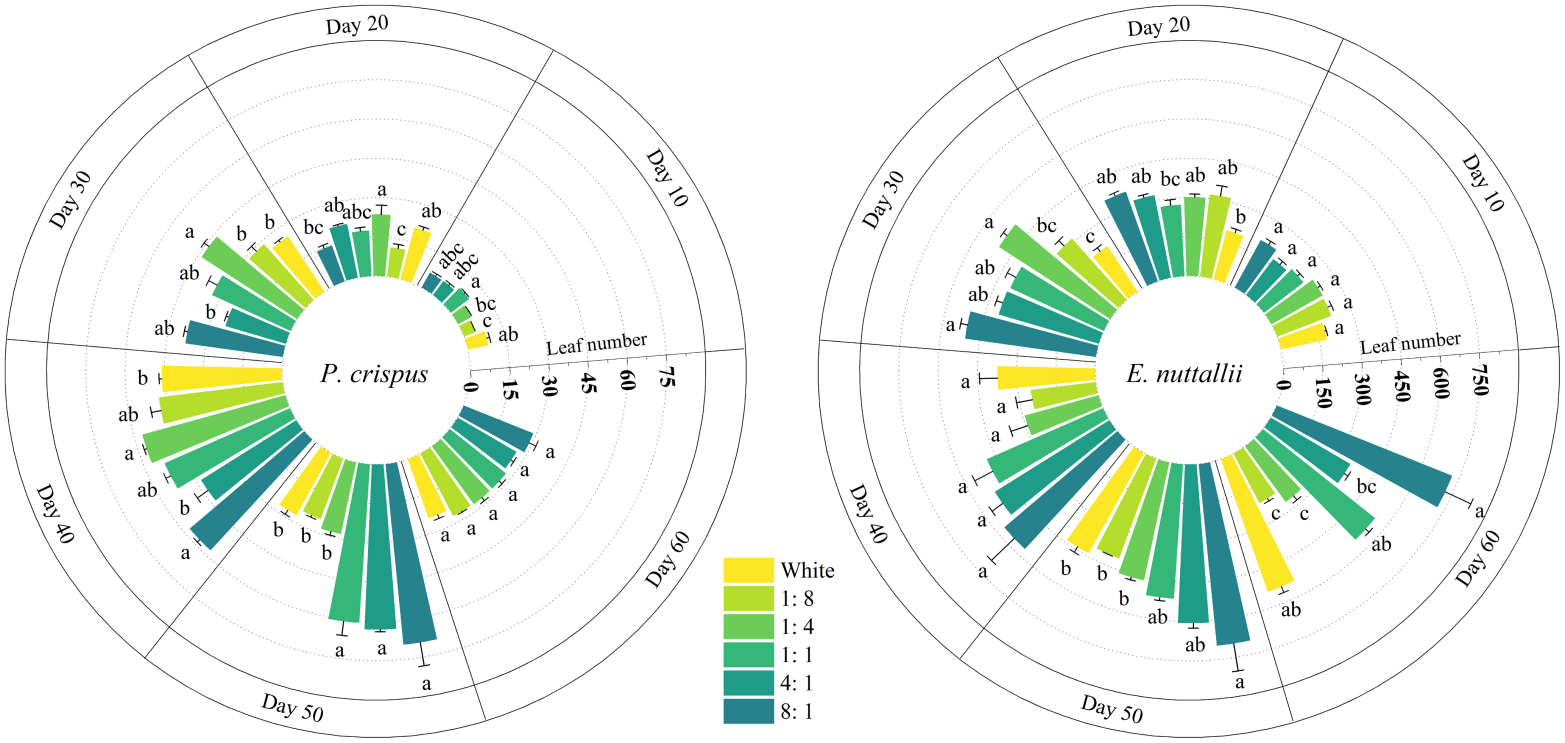
Effects of light quality on the leaf number of *P. crispus* and *E. nuttallii*.

Under white light, 1:8, and 1:4 light quality, the leaf numbers of *P. crispus* were the highest on day 40 (Figure 7), and then the leaves began to fall off. On day 50, the leaf numbers under these three light qualities were significantly lower than those of the other three treatment groups (*p* < 0.05).

Under more blue light (1:8, 1:4), leaves of *E. nuttallii* began to fall off after day 30 (Figure 7). At the end of the experiment, there were only 213.50 and 263.00 leaves, respectively, which were significantly lower than in the CK (*p* < 0.05). The leaf number of *E. nuttallii* under 8:1 light quality was the highest, at 721.67 pieces (*p* < 0.05).

#### 3.1.6 Leaf Length and Width

**FIGURE 8:**
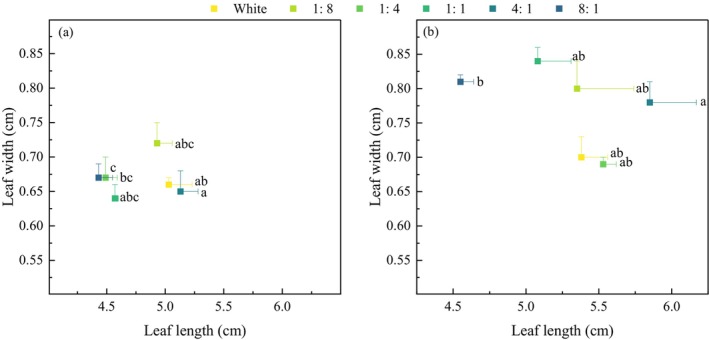
Effects of light quality on leaf length and width of *P. crispus*. (a) and (b) represent day 30 and day 60, respectively.

Light quality had little effect on leaf length and width of *P. crispus* (Figure 8). On day 30, only the 4:1 and 8:1 treatment groups showed significant differences in leaf length (*p* < 0.05). The leaf width varied between 0.64 and 0.72 cm (Figure 8a) (*p* > 0.05). At the end of the experiment, the leaf length in the 4:1 treatment group was longest (5.85 cm), whereas the leaf width was widest in the 1:1 treatment group (0.84 cm) (Figure 8b).

#### 3.1.7 Adventitious Root

**FIGURE 9:**
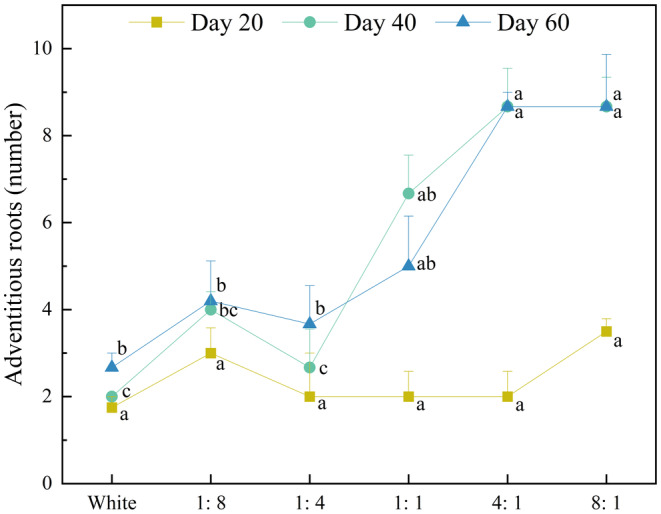
Effects of light quality on adventitious roots of *E. nuttallii*.

By day 20, there were no significant differences in the number of adventitious roots in *E. nuttallii* (*p* > 0.05) (Figure 9). After day 40, the number of adventitious roots began to differ significantly (*p* < 0.05). At the end of the experiment, adventitious roots in the 4:1 and 8:1 treatment groups were the most (8.67 in both groups) and those of the CK were the lowest (only 2.67). Overall, compared to under more red light (4:1, 8:1), *E. nuttallii* had lower adventitious roots under more blue light (1:4, 1:8).

#### 3.1.8 Root–Shoot Ratio

**FIGURE 10:**
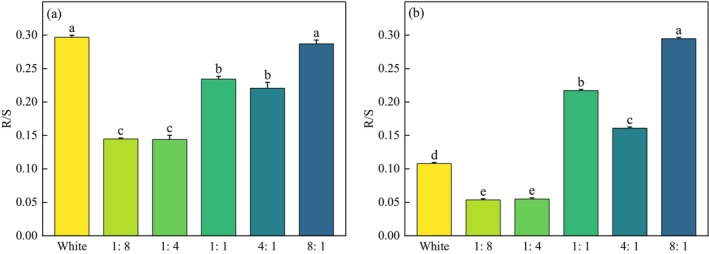
Effects of light quality on the root–shoot ratio of *P. crispus* (a) and *E. nuttallii* (b).

Under more blue light (1:4, 1:8), the R/S of *P. crispus* and *E. nuttallii* was smaller than under red light (4:1, 8:1) (Figure 10). The R/S under the different R/B treatment groups of *P. crispus* was lower than that of CK, while those of *E. nuttallii* were significantly higher than that of CK, except under the 1:4 and 1:8 light quality (*p* < 0.05).

### 3.2 Physiological Traits

#### 3.2.1 Photosynthetic Pigments

**FIGURE 11:**
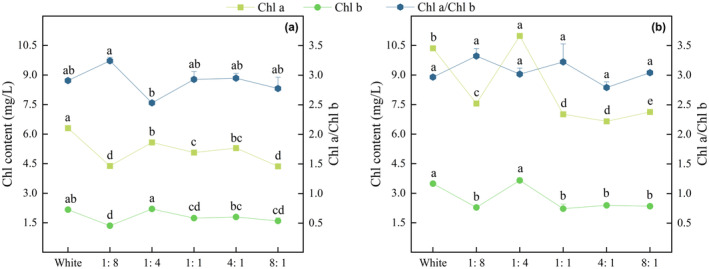
Effects of light quality on photosynthetic pigment contents of *P. crispus* (a) and *E. nuttallii* (b).

Under more blue light (1:8, 1:4), the Chl a, Chl b, and Chl a/Chl b of *P. crispus* and *E. nuttallii* were higher than those under red light (4:1, 8:1) (Figure 11). The Chl a of the two submerged macrophytes in the different R/B treatment groups (except 1:4) were lower than those in the CK. Under the same light quality, the Chl a and Chl b contents of *P. crispus* were lower than those of *E. nuttallii*. In contrast to photosynthetic pigments, the Chl a/Chl b ratio of *P. crispus* was the lowest under 1:4 light quality, whereas it was highest under 1:8 light quality. However, the effects of different light qualities on the Chl a/Chl b ratio in *E. nuttallii* were not significant (*p* > 0.05).

#### 3.2.2 MDA

**FIGURE 12:**
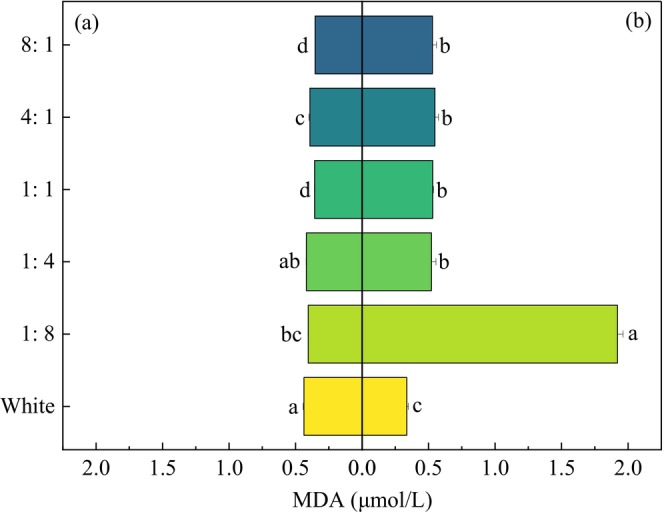
Effects of light quality on MDA in *P. crispus* (a) and *E. nuttallii* (b).

Under more blue light (1:4, 1:8), the MDA contents of the two submerged macrophytes were higher. However, the MDA content of *P. crispus* in the CK was the highest, whereas that of *E. nuttallii* was the lowest (*p* < 0.05) (Figure 12). The MDA content of *E. nuttallii* was higher than that of *P. crispus*, except under white light.

### 3.3 Stoichiometry

**FIGURE 13:**
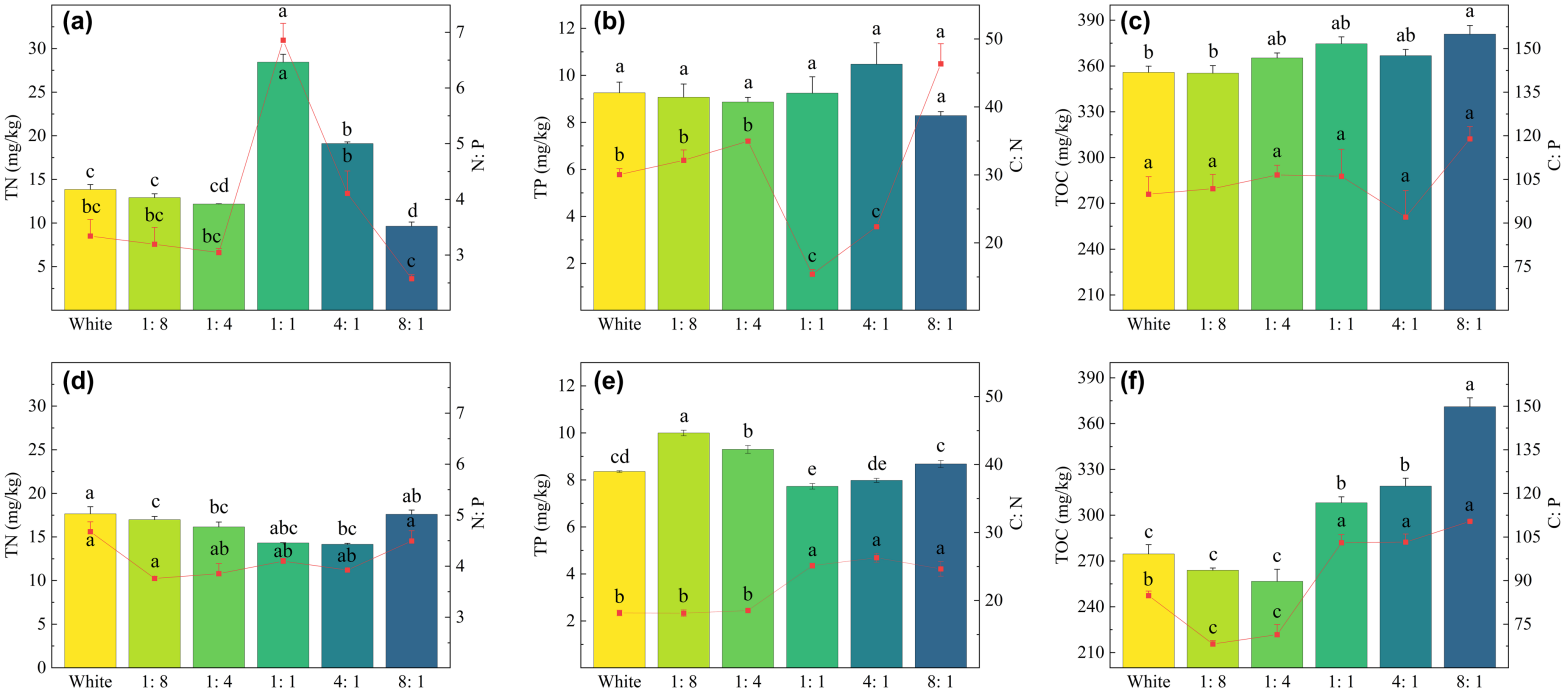
Effects of light quality on stoichiometry of *P. crispus* (a–c) and *E. nuttallii* (d–f). The bar chart represents the contents of TN, TP, and TOC, and the point‐line chart represents the N:P, C:N, and C:P ratios.

The TN, TP, and TOC contents in *P. crispus* and the N:P, C:N, and C:P ratios of *P. crispus* and *E. nuttallii* were higher under more red light (4:1, 8:1) than under more blue light (1:4, 1:8) (Figure 13). Under 1:1 light quality, the TN content (28.45 g/kg) and N:P ratio of *P. crispus* were the highest (*p* < 0.05), whereas those of *E. nuttallii* were lower and only slightly higher than under 4:1 light quality. Under 1:1 light quality, the C:N ratio of *P. crispus* was the lowest, which was significantly lower than that of the CK (*p* < 0.05).

In contrast to *P. crispus*, the TN and TP contents of *E. nuttallii* were higher under blue light (1:4, 1:8) than under red light (4:1, 8:1), whereas the TOC content showed the opposite trend. The TOC content and C:P ratio of *E. nuttallii* increased with increasing R/B ratios (*p* < 0.05).

## 4 Discussion

### 4.1 Effects of Light Quality on the Growth of *P. crispus* and *E. nuttallii*

Although *P. crispus* can propagate through its seeds and fragments, its seeds rarely germinate and the seed germination rate is only 0.001% under natural conditions (Rogers and Breeen 1980). Therefore, the reproduction of *P. crispus* primarily depends on turions. Water depth, temperature, substrate, turbidity, light intensity, salinity, and other factors affect the germination rate of *P. crispus* turions (Jian et al. 2001; Wang et al. 2006; Xia et al. 2020). Our study found that *P. crispus* under each light quality both begins to germinate on Day 2, because the initial growth of *P. crispus* seedlings mainly depends on the nutrients accumulated by their turions for autotrophic growth (Chen et al. 2006). Pan (2008) found that the germination rate of *P. crispus* was faster under red light, but slowest under blue light. In our study, *P. crispus* germinated the slowest under 4:1 light quality. This may be related to the light intensity and temperature. The light intensity of Pan (2008) was 578 lx with single red and blue lights and the germination time of turions was at the end of September, while our light intensity was 100 μmol/m^2^/s (about 2200–2300 lx) with different red and blue ratios, and the germination time was in early November when the temperature was lower.

In the middle of the experiment (Day 30), the plant height of *P. crispus* was significantly higher under the appropriate amounts of red light (8:1) and blue light (1:4) than under white light (*p* < 0.05). However, at the end of the experiment, the plant height of *P. crispus* under different R/B light qualities was lower than that of CK. After *P. crispus* grew to the water surface (45 cm) on Day 40, its growth rate began to slow and the change in plant height decreased. On the one hand, this may be related to the decrease of temperature. In the later stage of the experiment (Day 40–60), the average nighttime temperature was 4.34°C (Figure 2), which was very close to the survival ambient temperature (4 ± 1°C). However, in the last 20 days, the plant height of *P. crispus* in different R/B treatment groups was lower than that in the white light, indicating that long‐term red and blue light irradiation was not conducive to the growth of *P. crispus*. Wang et al. (2015, 2016) also found that with the extension of UV‐B radiation time, the plant height of *P. crispus* was significantly reduced, especially when *P. crispus* grew close to the water surface, and it finally died. However, in our experiment, *P. crispus* had least stem nodes under white light, and the node distance was larger. Under water depth stress, *P. crispus* captures more light by increasing node distance (Song et al. 2017). Although the water depth in our experiment was 45 cm, it did not cause stress to *P. crispus*; however, *P. crispus* may still increase the node distance to obtain more light to meet its growth needs. In contrast to *P. crispus*, different R/B lights were beneficial to the growth of *E. nuttallii*, such that their plant height (except under 1:4) was higher than that under white light, but there was no significant effect on stem nodes.

Previous studies have shown that, in shallow waters with low R/B ratios, submerged macrophytes tend to produce more sexual reproductive units, whereas in waters with a high ratio of red light, they produce more clonal propagules (Su et al. 2018). In our study, *P. crispus* and *E. nuttallii* also produced the most branches under red light. This was similar to *Vallisneria natans* (*V. natans*) and *Hydrilla verticillata* (*H. verticillata*) (Gao et al. 2023). However, *E. nuttallii* produced more branches than *P. crispus*, which may be due to their different growth forms. *P. crispus* is an erect submerged macrophyte that allocates more energy to upward upright growth (Oyaert, Volckaert, and Debergh et al. 1999); therefore, its branches are less than that of low‐canopy *E. nuttallii*. However, in the later stages of the experiment, the leaves of *P. crispus* fell off, resulting in no significant differences among the treatment groups (*p* > 0.05). In the present study, both *P. crispus* and *E. nuttallii* produced greater plant heights and more leaves, branches, and adventitious roots under more red light (4:1, 8:1), whereas under more blue light, both of them had low R/S, indicating that more blue light was not conducive to the accumulation of underground biomass of *P. crispus* and *E. nuttallii*.

Plant roots include main, lateral, and adventitious roots (Ye, Zhu, and Liao 2014). Adventitious roots enhance plant fixation and support plants, helping plants obtain nutrients and oxygen from water (Colmer 2003), and are also a way for plants to overcome adversity. Previous studies have shown that light affects the formation of adventitious roots in plants (Bai et al. 2020). Among submerged macrophytes, *E. nuttallii* and *H. verticillata* have adventitious roots. In our study, *E. nuttallii* grown under more blue light (1:4, 1:8) had more adventitious roots. After the adventitious roots were formed, when they were long enough to contact the sediment, they took root in the sediment, resulting in *E. nuttallii* tending to grow horizontally. Under white light, *E. nuttallii* tended to grow upright because of fewer branches and adventitious roots.

### 4.2 Effects of Light Quality on Physiological Indexes of *P. crispus* and *E. nuttallii*

Chlorophyll is the most important pigment in plant photosynthesis, which can measure the status of plant growth (Tian et al. 2023). Light quality can affect photosynthesis in plants (Wang et al. 2022). Under more red light, plants experience photosynthetic dysfunction (red light syndrome) (Li et al. 2017), which is manifested by insufficient photosynthetic C assimilation products and limited growth. In this experiment, more blue light (1:4, 1:8) was more conducive to the synthesis of photosynthetic pigments in the two submerged macrophytes. However, previous studies have found that the Chl content of *P. crispus* is the highest under red light, and that the photosynthetic efficiency of the leaves is the highest (Pan 2008). This may be due to differences in light intensity and quality. Light intensity affects the synthesis of photosynthetic pigments. Chl synthesis is blocked when light is insufficient and when the light intensity is too strong to promote the protection mechanism of plants, resulting in Chl decomposition or inhibition of photosynthetic pigment production (Chai et al. 2018). The light intensity used by Pan (2008) was 30% of that under natural light (3854 lx), and the light quality was controlled using a single blue and red filter film. Our experiment was conducted in a greenhouse under shade conditions. Our light intensity was 100 μmol/m^2^/s, and the light quality included different ratios of red and blue light. In terrestrial plants, such as *Lycoris radiata* and *Ginkgo biloba L*, a combination of red and blue light can significantly improve the growth rate and photosynthetic efficiency of plants, and the synthesis of photosynthetic pigments is greater than that under white light (Li et al. 2019; Yuan et al. 2023). Thus, the light quality with different R/B may be an important factor leading to differences in the results. In addition, in plants, Chl a and Chl b can be converted into each other to regulate photosynthesis. The smaller the Chl a/Chl b ratio, the stronger the light absorption capacity (Yuan, Hua, and An 2023). Only *P. crispus* in the 1:4 treament group has stronger light absorption capacity, while light quality had little effect on the light absorption capacity of *E. nuttallii* (*p* > 0.05).

During plant growth, stress can be reduced by regulating enzymes in the plant body. When the damage caused by external conditions to the cell membrane exceeds its defense ability, membrane lipid peroxidation is strengthened to produce MDA. The higher the MDA content, the more obvious the inhibition of plant growth and metabolism (Xia et al. 2020). In this experiment, the MDA of *P. crispus* in the different R/B treatment groups was lower than that in the white light, indicating that red and blue light caused less physiological stress on *P. crispus*. In *Ginkgo biloba* seedlings, the MDA content under white light was also higher than that under red, blue, and combination of red and blue light (Yuan et al. 2023). However, *E. nuttallii* showed the opposite trend, and the MDA of *E. nuttallii* was higher than that of *P. crispus*. The results showed that both red light and blue light caused greater damage to the metabolic activity of *E. nuttallii* compared to white light.

### 4.3 Effects of Light Quality on Stoichiometry of *P. crispus* and *E. nuttallii*

**FIGURE 14:**
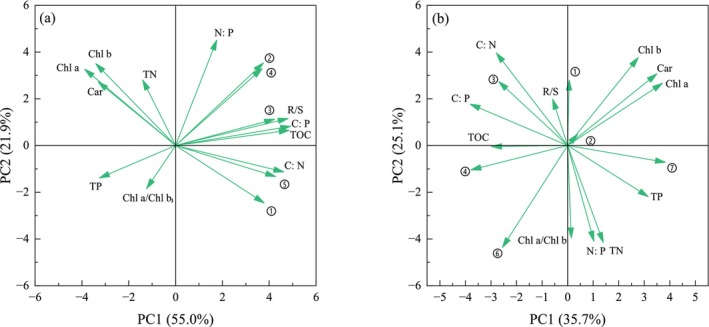
Results of principal component analysis. (a) *E. nuttallii*. (b) *P. crispus*. ① Plant height ② Stem node number ③ Branch number ④ Leaf number ⑤ Adventitious root ⑥ Leaf width ⑦ Leaf length.

C, N, and P are the most basic nutrients required for plant growth and are essential for physiological regulation (Reichert and Schuwirth 2010). C content is related to the C fixation rate of photosynthesis (Zhou, Wu, and Wang 2020). Different growth strategies lead to different C fixation rates in plants. *E. nuttallii* is a low‐canopy species. Under more red light, the TOC, C:N, and C:P contents in *E. nuttallii* were higher(Figure 13), indicating that red light promoted the absorption and utilization of N and P by *E. nuttallii*. The PC1 of *E. nuttallii* accounted for 55.0% of the total variance (Figure 14a). All morphological indicators had a high positive load on PC1, which also indicated that the growth of *E. nuttallii* was affected by the utilization efficiency of N and P (C:N, C:P). However, based on the number of stem nodes and leaves, the relationship between the two and photosynthetic pigments was stronger because more leaves were conducive to obtaining more light and promoting the accumulation of photosynthetic pigments. *P. crispus* is an erect submerged macrophyte that can grow on water surfaces. Therefore, under different R/B ratios light qualities, the TOC content of each treatment group of *P. crispus* was higher than that of the CK, and the C fixation rate of photosynthesis was greater than that of the low‐canopy *E. nuttallii* growing underwater. The N:P ratios of *P. crispus* and *E. nuttallii* were less than 14 under each light quality, indicating that their growth was limited by N. PC1 and PC2 of *P. crispus* accounted for 35.7% and 25.1% of the total variance, respectively (Figure 14b). Leaf length had a higher positive load on PC1, which was closely related to the two photosynthetic pigments and N and P content, whereas leaf width was opposite to leaf length, which may be related to the fact that *P. crispus* obtains more light by extending leaf length. PC2 was dominated by plant height and branch number, and the accumulation of photosynthetic pigments and high N‐ and P‐utilization efficiencies promoted the upward growth and branching of *P. crispus*.

### 4.4 Implications for the Management of Submerged Macrophytes

The turions of *P. crispus* germinated well under different light qualities. Thus, during ecological restoration, turions can be fully utilized for reproduction. Our results contradicted our hypothesis. As an erect submerged plant, *P. crispus* did not grow better more under blue light. Both *P. crispus* and *E. nuttallii* grew better under more red light (4:1, 8:1), which showed higher plant heights and more leaves, branches, and adventitious roots under red light (4:1, 8:1). Therefore, *P. crispus* and *E. nuttallii* can better adapt to the light environment of eutrophic water dominated by red light. However, under more blue light, *P. crispus* and *E. nuttallii* were more stressed, and excessive blue light was not conducive to the synthesis of photosynthetic pigments in *E. nuttallii*. When water quality improves owing to the reduction in suspended particulate matter and phytoplankton, blue light has a stronger penetration ability in the water. Compared to red light, excessive blue light may not be conducive to the growth of *P. crispus* and *E. nuttallii*. Therefore, when using *P. crispus* and *E. nuttallii* to improve water quality, it is necessary to supplement them with other evergreen or overwintering submerged macrophytes (such as *Vallisneria spinulosa*) so that the clear water state of the lake can be maintained for a long time (Wang et al. 2024; Zhao et al. 2023). However, it cannot be ignored that turbidity, including biogenic and non‐biogenic turbidity (Ma, Li, and Bian 2021), plays a key role in the absorption and scattering of light, which can affect the attenuation of light (Ibelings et al. 2007; Lin et al. 2024) and then affect the absorption efficiency of light by submerged macrophytes. The increase of turbidity is an important reason for the disappearance of submerged macrophytes and the regime shift from grass‐type lakes to algae‐type lakes in the process of lake eutrophication (Wang et al. 2021). It is regretful that our study lacks tracking observation of water turbidity, so in the follow‐up study, it can strengthen the study of turbidity.

## 5 Conclusion

Differences in the morphological, physiological traits, and stoichiometry of *P. crispus* and *E. nuttallii* under six light qualities were studied using a controlled experiment. The conclusions are as follows:

1. The germination number of turions of *P. crispus* increased with the increase of the R/B, but there were no significant differences in the germination rate and leaf length and width under different light qualities (*p* > 0.05).
2. Both *P. crispus* and *E. nuttallii* produced greater plant height, more leaves and branches, adventitious roots, and R/S under more red light (4:1, 8:1). White light was not conducive to producing branches and adventitious roots of submerged plants.
3. Compared with more red light, more blue light was more conducive to the synthesis of photosynthetic pigments. However, under more blue light, both *P. crispus* and *E. nuttallii* were subjected to strong physiological stress.
4. Red light was more beneficial for the synthesis of TN, TP, and TOC in *P. crispus*, and the N:P, C:N, and C:P ratios of the two submerged macrophytes were higher. However, the TN and TP contents of *E. nuttallii* were higher under more blue light.

Both *P. crispus* and *E. nuttallii* can better adapt to the light environment of eutrophic waters dominated by red light; however, when used for aquatic restoration, they need to be supplemented with other submerged macrophytes after water quality improvement.

## Author Contributions

**Xiaowen Lin:** conceptualization (equal), data curation (equal), formal analysis (equal), investigation (equal), methodology (equal), validation (equal), visualization (equal), writing – original draft (equal). **Xiaodong Wu:** conceptualization (equal), funding acquisition (equal), methodology (equal), writing – review and editing (equal). **Xuguang Ge:** funding acquisition (equal), project administration (equal), supervision (equal). **Chenxin Zhong:** data curation (equal), investigation (equal), methodology (equal). **Zian Xiang:** data curation (equal), investigation (equal). **Ye Yao:** software (equal), visualization (equal). **Lishuai Zhang:** visualization (equal). **Sizhuo Li:** validation (equal).

## Conflicts of Interest

The authors declare no conflicts of interest.

## Supporting information


Appendix S1

## Data Availability

The datasets for this study are accessible in Appendix S1.
